# Denitrative Cross-Couplings of Nitrostyrenes

**DOI:** 10.3390/molecules25153390

**Published:** 2020-07-27

**Authors:** Michaela Marčeková, Branislav Ferko, Katarína Ráchel Detková, Pavol Jakubec

**Affiliations:** Faculty of Chemical and Food Technology, Slovak University of Technology, Radlinského 9, 81237 Bratislava, Slovakia; michaela.marcekova@stuba.sk (M.M.); branislav.ferko@stuba.sk (B.F.); xdetkovak@stuba.sk (K.R.D.)

**Keywords:** stereoselective, nitrostyrene, cross-coupling, radical, photochemical, chalcone, stilbene, conjugated, sulfone, phosphonate

## Abstract

Interestingly, β-nitrostyrenes, typically bench stable compounds, are highly promising cross-coupling partners, due to their excellent availability and well understood reactivity. In this review, we report on the discovery and advancements, in the field of stereoselective, denitrative cross-couplings of β-nitrostyrenes with miscellaneous organic reagents. The rapidly expanding field offers alternative access to a broad range of functionalized alkenes, including β-alkylated styrenes, chalcones, stilbenes, cinnamic acids, and conjugated sulfones and phosphonates. The most important mechanistic pathways are briefly discussed, to familiarize readers with the elementary reactions occurring during the coupling.

## 1. Introduction

Cross-couplings in modern organic synthesis have largely focused on noble transition metal-catalyzed reactions of privileged functional groups. The Suzuki, Heck, Stille and Sonogashira reactions have become irreplaceable tools of the modern era organic synthesis. However, the low abundance of the required transition metals and the demand for greener and more economical transformations have led to the development of more sustainable cross-couplings employing unconventional functional groups. The nitro group represents one of the most versatile functional groups in organic chemistry. Organic compounds containing the nitro group offer such a huge variety of reactivity patterns and open access to so many different products, that the nitro group gained a nickname, “synthetic chameleon” [1,2]. Arguably, the most utilized classes of nitro compounds, distinctive in their reactivity, include nitroalkanes, nitroarenes, and nitroalkenes. Nitroalkanes, due to the high Brønstedt acidity at the α-position, found endless applications as powerful pro-nucleophiles in different variants of the nitro-Mannich (*aza*-Henry) [3], the Henry reaction (nitro-aldol) [4,5] and the conjugate addition [6]. Direct transformations of the nitro group in nitroalkanes have been firmly established as highly valuable tools of organic synthesis. Reductions of the nitro group [7], proto-denitration reactions [8,9,10,11], and the Nef reaction [12] belong to the most powerful transformations of nitroalkanes. Significantly different reactivities of nitroarenes and nitroalkenes are determined by the direct bonding of the nitro moiety to a sp^2^-hybridised carbon atom. Nitroarenes serve not just as irreplaceable raw materials for the modern era materials manufacturing, but also as text-book examples to demonstrate fundamental principles of organic chemistry—aromatic electrophilic and nucleophilic substitution. Related nitroalkenes, and especially nitrostyrenes, have also been established as essential and highly popular building blocks of organic synthesis. Their straightforward synthesis is enabled via the reliable Henry condensation—dehydration sequence from readily available aldehydes and nitroalkanes [13]. Multiple alternatives, such as the direct nitration of alkenes and the oxidation of enamines, have emerged as complementary synthetic protocols [14]. Typically bench-stable solids, they serve as potent dienes and dienophiles in Diels–Alder reactions [14]. However, perhaps the most important reason for nitrostyrenes’ popularity is their well-documented reactivity as powerful electrophiles with multiple reaction centers, especially in conjugate additions [15]. Both the excellent accessibility and the characteristic reactivity of nitrostyrenes contributed to the discovery and rapid development of a new class of their transformation—denitrative cross-couplings. The novel denitrative cross-couplings of nitrostyrenes are the major topic of this review [16].

## 2. Nitrostyrene Cross-Coupling—The Background and Mechanisms

**Scheme 1:**
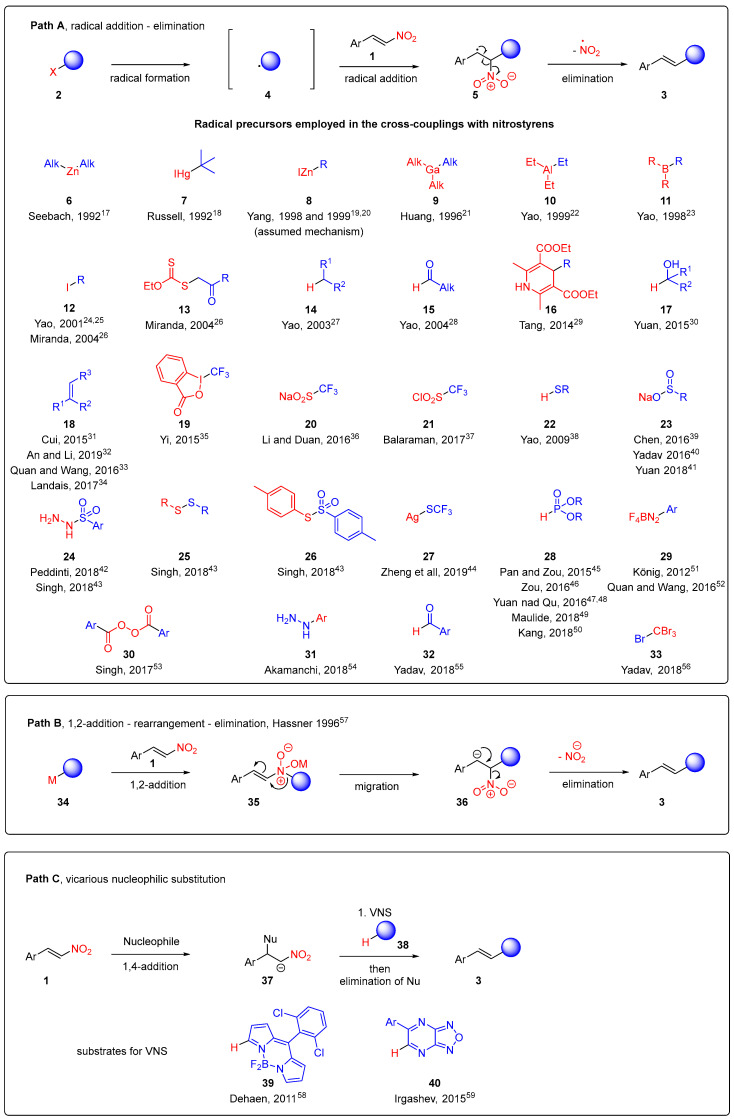
Denitrative cross-couplings of nitrostyrene—mechanistic proposals [17,18,19,20,21,22,23,24,25,26,27,28,29,30,31,32,33,34,35,36,37,38,39,40,41,42,43,44,45,46,47,48,49,50,51,52,53,54,55,56,57,58,59].

**Scheme 2:**
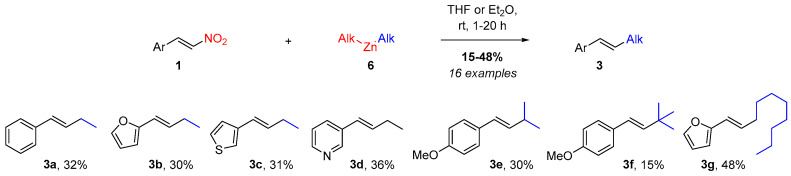
Seebach’s ground-breaking discovery of novel Et_2_Zn reactivity with nitrostyrene [17].

**Scheme 3:**
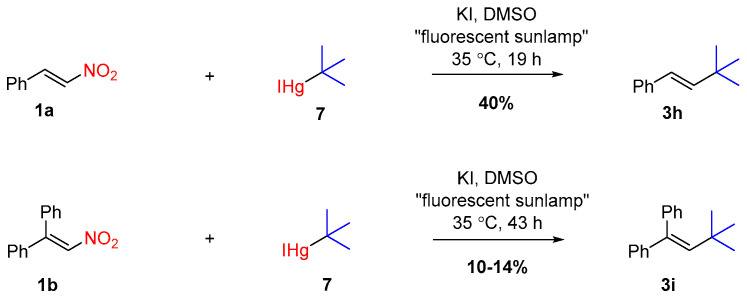
Reactivity of organomercury compounds with nitrostyrenes described by Russell [18].

Denitrative cross-couplings allow the formation of a new bond between the styryl part of nitrostyrene **1** and the cross-coupling partner **2** yielding alkene **3** (Scheme 1). The reaction is accompanied by the simultaneous elimination of a small molecule containing NO_2_ residue. To the best of our knowledge, the first transformations of this kind were independently reported by Seebach and Russell in 1992 (Scheme 2 and Scheme 3) [17,18]. Ever since, the transformation rapidly grew into an emerging field of organic synthesis, providing an exciting alternative to the traditional cross-couplings. The intensified research, especially in the last decade, culminated in the development of almost 50 unique methodologies, enabling the formation of C-C and C-heteroatom bonds with the styryl residue (Scheme 1).

Three recognizable mechanistic scenarios have been proposed to rationalize the intriguing transformation. These include a radical addition-elimination path A, organometallic addition-migration-elimination path B, and vicarious nucleophilic substitution path C (Scheme 1). The radical addition-elimination route A is the most frequently proposed mechanistic path of the nitroolefin cross-coupling. Although each proposal differs in detail, the main three characteristic steps feature in all proposals. Firstly, carbon- or heteroatom-centered radical **4** is generated in situ by various methods from precursors **6**–**33** [17,18,19,20,21,22,23,24,25,26,27,28,29,30,31,32,33,34,35,36,37,38,39,40,41,42,43,44,45,46,47,48,49,50,51,52,53,54,55,56]. These include a thermal homolytic cleavage of labile bonds, metal-catalyzed and photochemically initiated processes. Then, the in situ generated radical **4** undergoes a regioselective radical addition to the nitroolefin **1**, giving rise to the C-centered benzylic radical **5**, which, upon the spontaneous elimination on the nitrosyl radical, yields the coupling product **3**. Path B begins with a regioselective 1,2-addition of organometallics to nitroolefins, producing intermediate **35**, which upon migration and the following elimination of the nitrate anion yields alkene **3**. Hassner proposed such unique mechanism for organomanganese derivatives [57]. Path C involves the initial conjugate addition of a nucleophile to nitrostyrene **1** yielding nitronate **37**, which, after the sequential vicarious nucleophilic substitution and elimination, gives alkene **3**. Path C is possible only for cross-coupling partners with special structural features enabling the VNS [58,59].

## 3. Nitrostyrenes in Denitrative C(sp^2^)-C(sp^3^) Cross-Couplings

During the investigation of enantioselective Michael additions of diethylzinc to nitrostyrenes, Seebach made a surprising discovery [17]. Instead of the expected 1,4-addition, he observed the formation of 1-phenylbut-1-ene (3a), the product of the substitution of the vinylic nitro group by ethyl group (Scheme 2). The substitution was preferred over the 1,4-addition in the absence of a Lewis acid, and in ethereal solvents such as tetrahydrofuran and diethyl ether. The groundbreaking discovery allows for the formation of *trans*-substituted styrenes 3 containing ethyl, isopropyl, *tert*-butyl, and octyl substituents. Despite the moderate chemical yields, this pioneering work opened utterly new horizons of organic chemistry, and disclosed an unprecedented reactivity pattern of nitrostyrenes, with huge synthetic potential. 

At the same time, independently from Seebach, Russell discovered the analogous behavior of organomercury compounds [18]. When nitrostyrenes 1a and 1b were treated with *tert*-butylmercury iodide (7) in the presence of potassium iodide under irradiation, β-*tert*-butylstyrenes 3h and 3i were formed in moderate yields (Scheme 3). According to the proposed mechanistic justification, the transformation involved the *tert*-butyl radical formation, followed by a radical addition/elimination sequence. Only two specific examples were reported. 

**Scheme 4:**
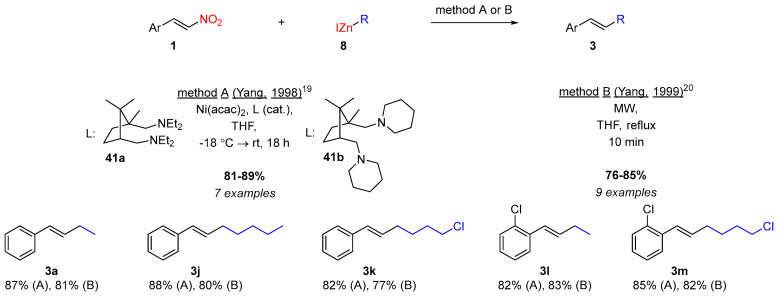
Yang’s substitution of the nitro group in nitrostyrene by organozinc reagents [19,20].

A related cross-coupling of organometallics with nitroolefins 1 was reported in 1998. Yang described simple organozinc iodides 8 taking part in a nickel-catalysed denitrative formation of alkenes 3 (Scheme 4, method A) [19]. The mechanistically unexplored but intriguing transformation was catalyzed by a complex formed in situ from nickel acetate and tertiary amine 41a. The alternative catalytic system formed from nickel acetate and ligand 41b or triethylamine performed almost equally well (the yields reported in Scheme 4 refer to the catalytic system using ligand 41a). Although the described substrate scope was relatively narrow, seven examples were reported, and the reaction proceeded smoothly at ambient temperature, with excellent chemical yields, ranging from 81 to 89%. A year later, in 1999, the same research group reported a modified variant of the coupling [20]. Simple and functionalized organozinc iodides underwent the cross-coupling under microwave irradiation. The absence of any additional catalyst and very attractive, short reaction times are the most remarkable features of the second protocol (Scheme 4, method B). 

**Scheme 5:**
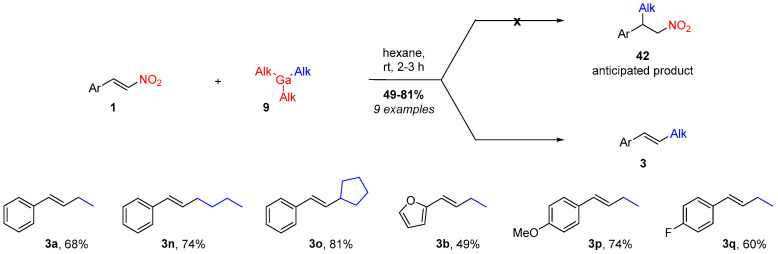
Huang’s denitrative cross-coupling of nitrostyrene and alkylgallium compounds [21].

In both firstly reported cross-couplings of nitrostyrenes, successfully reacted organometallics were derived from elements of the 12th column of the periodic table (Scheme 2 and Scheme 3). A similar reactivity with nitrostyrenes was observed for multiple organometallics derived from the related elements located in the 13th column of the table—gallium, aluminum, and boron. The unexpected reactivity of trialkylgallium **9** in the reaction with nitrostyrene **1** was observed by Huang [21]. Instead of the anticipated conjugate addition to the nitrostyrene **1** and the formation of adduct **42**, the authors noted a smooth conversion to alkyl-substituted styrenes **3** (Scheme 5). A variety of trialkylgallium compounds easily accessible from the corresponding Grignard reagents underwent the transformation in up to 81% yield. Based on several experiments, a radical addition-elimination mechanism was proposed to explain the unexpected reactivity (Scheme 1, Path A). 

**Scheme 6:**
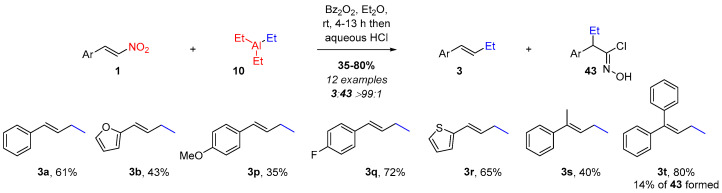
Substitution of the nitro group in nitrostyrenes by triethylaluminum reported by Yao [22].

**Scheme 7:**
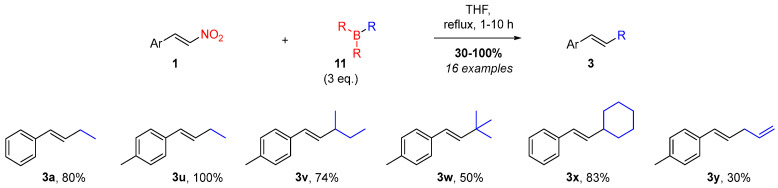
Yao’s cross-coupling of nitrostyrenes and trialkyl and triallylboranes [23].

Only a few years after Huang’s discovery, Yao investigated a reaction of nitrostyrenes **1** with triethylaluminium (**10**) [22]. The reactions were performed in diethyl ether solution, under inert gas, and depending on the exact conditions, generated either alkenes **3** or hydroximoyl chlorides **43** or both (Scheme 6). When triethylaluminum was used in combination with a stoichiometric amount of dibenzoyl peroxide, the reaction produced exclusively alkenes **3** (**3**:**43** >99:1). An exception was 2-phenyl substituted nitrostyrene, which also yielded a small amount of hydroximoyl chloride **43**. The transformation was proposed to proceed as a free-radical reaction. The claim was supported by an observation that the yields of alkenes **3** were increased in the presence of benzoyl peroxide and decreased in the presence of galvinoxyl, a known radical scavenger. Several reaction parameters, including the nature of the aluminum cross-coupling partner, the presence of an additional Lewis acid, and UV-light irradiation, significantly influenced the ratio of alkene **3** and hydroximoyl chloride **43**. A closely related reaction of nitrostyrenes **1** with trisubstituted boranes **11** producing alkylated styrenes **3** was studied in 1998 by Yao (Scheme 7) [23].

The exploration of the reaction scope mostly involved a variation of the nitrostyrenes **1**; the trisubstituted boranes **11** were examined to a lesser extent. Compare to the broad range of aryl substituents in nitrostyrene, only five readily available organoboranes successfully converted to the corresponding sets of alkenes containing ethyl, *sec*-butyl, *tert*-butyl, cyclohexyl and allyl substituents. Although the transformation requires three-fold excess of the organoboron reagents, the short reaction time and good access to both starting materials **1** and **11** allowed straightforward and stereoselective access to *E*-configured olefins **3**.

**Scheme 8:**
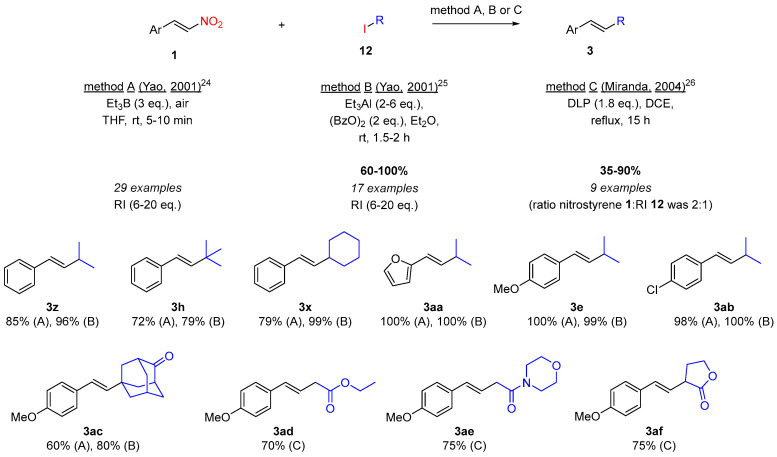
Yao’s and Miranda’s cross-coupling of nitrostyrenes and alkyl iodides [24,25,26].

Within the next few years, Yao’s research group reported several variations and improvements of the trialkylborane coupling. Yao’s enhancement reported in 2001 took advantage of the abilities of trialkylborane to promote the alkyl radical formation from alkyl iodides. Thus, various alkyl iodides **12** reacted with nitrostyrenes **1** in the presence of triethylborane, producing alkenes **3** under very mild conditions, at an impressive reaction rate (Scheme 8, method A) [24]. Due to the better availability of alkyl iodides, such modification of the original reaction conditions allowed a significant expansion of the original method scope. Altogether, twenty-nine substituted styrenes **3** were formed by the new method. Nevertheless, the necessity to utilize a substantial excess of both the alkyl iodide (6–20 equivalents) and triethylborane (3 equivalents) still could be viewed as a limiting aspect of the otherwise admirable method. 

In 2001, Yao demonstrated that the cross-coupling of nitrostyrenes **1** with alkyl iodides **12** could also be promoted by triethylaluminum (Scheme 8, method B) [25]. Like the trialkylborane promoted cross-coupling, the method employed several fold-excess of triethylaluminum (2–6 eq.) and alkyl iodide (6–20 eq.), alongside with two-fold excess of dibenzoyl peroxide. Another cross-coupling of nitrostyrenes with organic iodides was reported in 2004. Miranda generated stabilized radicals from the corresponding iodides, using DLP as a radical initiator (Scheme 8, method C) [26]. The radical formation was initiated by the thermal decomposition of the peroxide. The noteworthy feature of the method is undoubtedly the higher structural complexity of the coupling partners. Iodides bearing the oxo group and ester and lactone moieties were utilized in the transformation, providing straightforward access to non-conjugated carbonyl compounds **3ad**, **3ae** and **3af**. Another distinctive characteristic is the employment of a slightly smaller excess of the radical initiator. Moreover, 1.8-fold excess of DLP compared to nitrostyrene **1** was used.

**Scheme 9:**
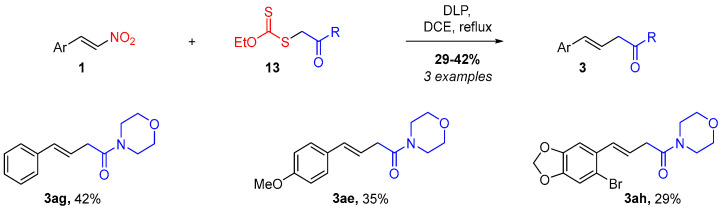
Miranda’s cross-coupling of xanthate and nitrostyrenes [26].

Miranda’s attempt to extend the attractive iodide coupling to readily accessible xanthates **13** was hampered by an unexpected low conversion, and a lack of understanding thereof (Scheme 9) [26]. Despite the attempted optimization, only low conversion and the moderate yields were obtained. 

**Scheme 10:**
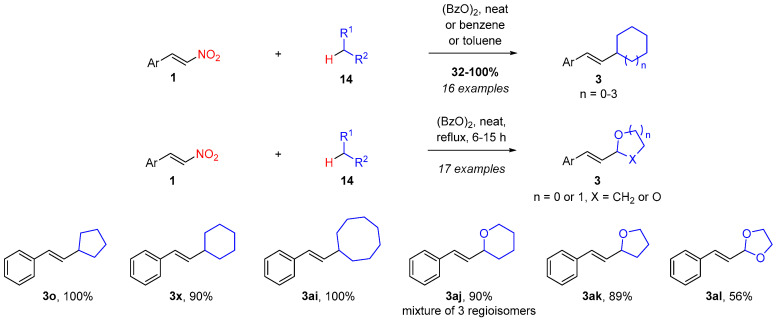
Yao’s C-H activation in cycloalkanes and cyclic ethers in reactions with nitrostyrenes [27].

**Scheme 11:**
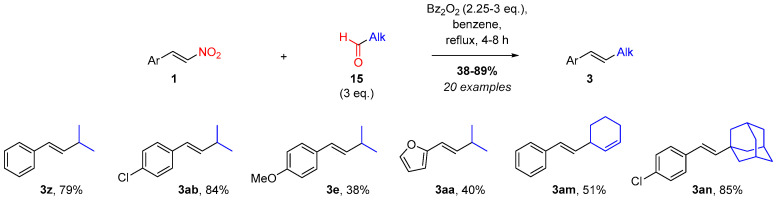
Yao’s denitrative, decarbonylative cross-coupling of nitrostyrenes and aromatic aldehydes [28].

In another report, Yao’s group exploited unfunctionalized alkanes and cyclic ethers **14**, as partners for the denitrative cross-coupling (Scheme 10) [27]. This strategy employed different alkyl radicals generated directly from cycloalkane and cyclic esters via C-H abstraction. Unlike in the previous Yao’s reports (Scheme 7 and Scheme 8), where triethylborane was employed as the initiator, benzoyl peroxide was used to enable the radical reaction. The substitution of the nitro group for cycloalkanes was successfully achieved with 5-, 6-, 7-, and 8-membered cycles, with various aryl substituents at the nitrostyrenes **1**. Excellent regioselectivity of the C-H functionalization of tetrahydrofuran and dioxolane was observed; only exclusive isomers **3ak** and **3al** were isolated. On the other hand, a mixture of all three possible regioisomers **3aj** was formed in the reaction with tetrahydropyran. Such a remarkable difference was justified by the kinetics of the H-abstraction and the preeminent radical trapping ability of nitrostyrene. Dibenzoyl peroxide proved to be a highly reliable radical initiator in a cross-coupling between nitrostyrenes **1** and aromatic aldehydes **15**, discovered by Yao (Scheme 11) [28]. The initially formed acyl radical (not shown) underwent fragmentation by extrusion of CO, producing a highly reactive alkyl radical. This intermediate reacted in refluxing benzene with substituted nitrostyrenes **1**, and produced a range of alkylated styrenes **3**.

Apart from the successful examples, Yao also reported a significant limitation; primary aldehydes such as propanal and valeraldehyde failed to yield the expected alkenes. Similar to the several proceeding pioneering couplings, the method utilized the excess of the coupling partner (3 equivalents) and the initiator (2–3 equivalents). 

**Scheme 12:**
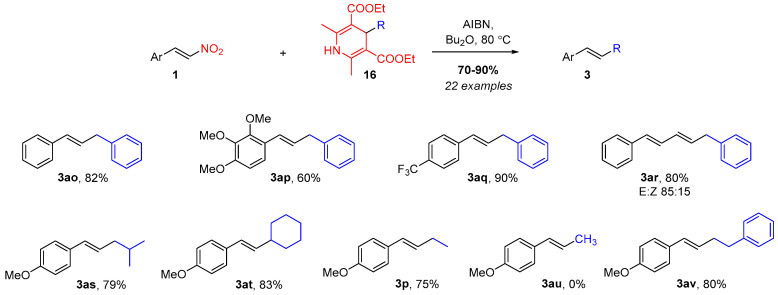
Tang’s utilization of Hantzsch esters in cross-coupling with nitrostyrenes [29].

A different combination of the radical precursor and initiator for a cross-coupling with nitrostyrenes **1** was reported by Tang in 2014 [29]. A reaction between nitrostyrene **1** and various Hantzsch esters **16** performed in hot dibutyl ether initiated by AIBN allowed the synthesis of a broad range of β-substituted styrenes **3** (Scheme 12). The investigation into the reaction scope revealed a huge variety of nitrostyrenes **1** and Hantzsch esters **16**. On the other hand, also certain limitations of the reaction scope were identified. Both substrates containing a substituent at the beta position and the nitro group at the aromatic ring failed to yield any desired product (not shown). It was attributed to an inhibiting effect of the nitro group and a steric hindrance. Several other noteworthy features are associated with the methodology. Apart from the unique production of alkyl radicals by a C-C bond cleavage, the method is characterized by a high degree of diversity of transferred alkyl groups, including benzyl, secondary, and primary alkyl groups. Especially the latter happened to be challenging for many other cross-couplings of nitrostyrene. No reaction times are given in the report. 

**Scheme 13:**
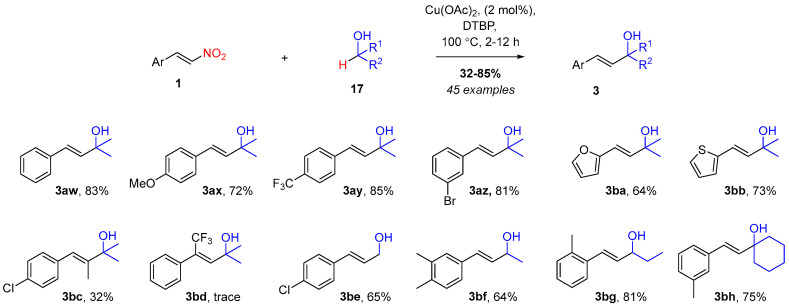
Yuan’s alkenylation of alcohols with β-nitrostyrenes [30].

An alkenylation of alcohols with β-nitrostyrenes **1** via a radical addition–elimination process was discovered by Yuan in 2015 (Scheme 13) [30]. The copper acetate catalyzed process performed at a very high temperature enabled the formation of miscellaneous allylic alcohols **3**, from a broad range of readily available primary and secondary alcohols **17**. The methodology holds a special position amongst the related couplings due to the vast variety of the coupling partners. Almost fifty allylic alcohols were prepared to demonstrate the broad scope. On the other hand, several structurally specific alcohols and β-alkyl substituted nitroolefins were not suitable for the coupling. Thus, allyl alcohol, phenylmethanol, and *tert*-butanol did not give the desired product. 

**Scheme 14:**
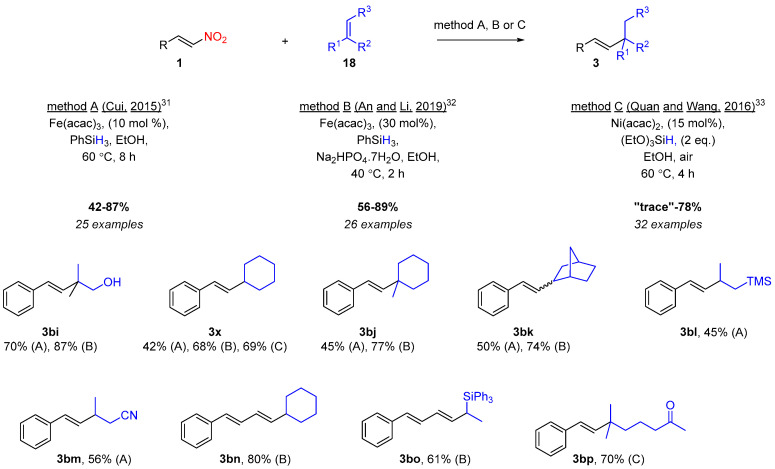
Denitrative cross-coupling of nitroalkenes and inactivated alkenes [31,32,33].

Due to their excellent commercial availability and natural abundance, unactivated alkenes are the hugely popular feedstock of organic synthesis. Their first denitrative coupling with β-nitroalkenes allowing access to valuable alkylated styrenes was developed by Cui in 2015 (Scheme 14, method A) [31]. The iron-catalyzed reaction proceeds through a radical pathway, with β-nitroalkenes serving as the vinylating reagents. Remarkable is the functional group tolerance, trimethylsilyl-, cyano- and hydroxyl moieties remained intact during the transformation to functionalized alkene **3**. The idea of denitrative alkylation of nitrostyrenes with alkenes was developed further by An and Li in 2019, when the scope was expanded to conjugated nitrodienes (Scheme 14, method B, R in **1** is ArCH=CH-) [32]. The addition of sodium hydrogen phosphate to Cui’s catalytic system enabled the straightforward formation of dienes, such as **3bm** and **3bn**. Moreover, An and Li applied the modified catalytic system doped with sodium hydrogen phosphate in the coupling with β-nitrostyrenes. The higher isolated chemical yields of alkenes **3bi**, **3x, 3bj** and **3bk** indicated an improvement of Cui’s catalytic system. It was justified by the in situ generation of the active silane PhSi(OEt)H_2,_ which prevented the deactivation of the catalyst.

**Scheme 15:**
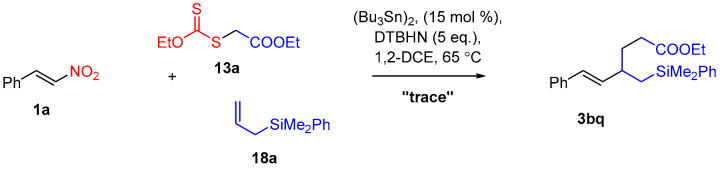
Multicomponent coupling of nitrostyrene with unactivated alkene and xanthate reported by Landais [34].

A closely related transition metal-catalyzed reductive cross-coupling of nitrostyrenes with unactivated alkene was also reported by Quan and Wang (Scheme 14, method C) [33]. Their novel catalytic system included a nickel-complex instead of the previously reported iron-complex. Compared to the Cui’s system, another marginal difference was reported for the silane-based reducing agent; triethoxysilane turned out to be the most efficient source of hydrogen. On the other hand, a resemblance to the Cui’s system is visible in the reaction solvent and temperature. In 2017, Landais spotted a rare multicomponent coupling of nitrostyrenes with an alkene [34]. Apart from nitrostyrene **1a** and alkene **18a**, the 3-component coupling involved xanthate **13a** (Scheme 15). Landais investigated a multicomponent reaction between xanthates, alkene and various Michael acceptors, such as acrylates (not shown). Nitrostyrene **1a,** as powerful Michael acceptor, was found unreactive under standard conditions, and only a trace amount of coupling product **3bq** was observed. Nevertheless, even the detected trace amount of silane **3bq** provided a proof of concept that nitrostyrenes could undergo a multicomponent transformation with alkenes. The one-pot protocol enabled the formation of two new C-C sigma bonds.

**Scheme 16:**
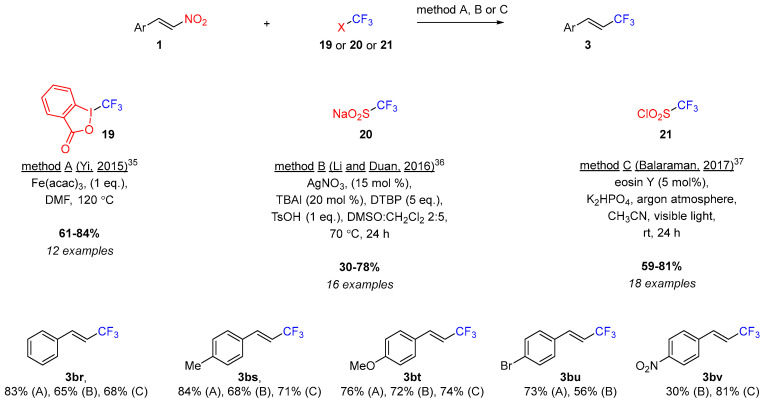
Denitrative trifluoromethylation using various available trifluoromethylating reagents [35,36,37].

Trifluoromethylation has recently emerged as a rapidly evolving method of modern organic synthesis. The late-stage modification of organic molecules with trifluoromethyl functionality became a popular and irreplaceable tool broadly applicable in various branches of industrial chemistry [60]. In the past decades, a plethora of useful synthetic methods alongside various practical reagents has been developed. Some of the reagents have been very recently applied in direct denitrative trifluoromethylations of nitrostyrenes (Scheme 16). The pioneering work was performed by Yi, who used Togni’s (II) reagent (**19**) in combination with iron(III) acetylacetonate at a high temperature, to promote the reaction (Scheme 16, method A) [35]. Both Togni’s reagent (**19**) and the iron complex were used in stoichiometric amounts. The denitrative trifluoromethylation tolerated miscellaneous substituents on the aromatic ring of nitroolefins. These include electron-donating and withdrawing—functional group and halogen atoms—even substrates containing heterocycles, and a substituent at the β-position underwent the transformation with respectable chemical yields. 

In 2016, Li and Duan developed an alternative protocol for the denitrative trifluoromethylation (Scheme 16, method B) [36]. Commercially available Langlois reagent (**20**), sodium salt of trifluoromethanesulfonic acid, served as a different source of the trifluoromethyl radical. After an extended optimization, the authors identified a cocktail of reagents necessary for successful trifluoromethylation. Apart from the oxidants, it contained TBAI and PTSA as the additives. Already, in 2017, a metal-free denitrative trifluoromethylation using trifluoromethansulfonyl chloride (**21**) was developed by Balaraman (Scheme 16, method C) [37]. The photochemical process catalyzed by eosin-Y operated at room temperature in acetonitrile under irradiation with daylight. The coupling is exceptional for its environmentally benign character and very mild reaction conditions. Moreover, apart from the attractive mild reaction conditions, the methodology offers a broad substrate scope. 

**Scheme 17:**
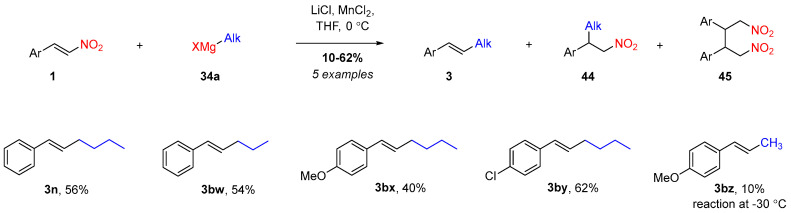
Hassner’s denitrative cross-coupling of nitrostyrene and organomanganese derivatives [57].

The most common mechanistic route in the cross-coupling of nitrostyrenes involves the addition of an in situ generated radical to nitrostyrene **1**, resulting in a formation of a benzylic radical, followed by elimination to restore the double bond (Scheme 1, path A). Such a mechanistic scenario has been proposed and supported by experimental evidence by several research groups. A different mechanistic pathway for the nitrostyrene cross-coupling was reported in 1996. Relying on the electrophilic nature and multiple reaction centers of nitrostyrenes **1**, Hassner exploited their high reactivity towards various nucleophilic organometallics [57]. Particularly, a reactivity of in situ formed organomanganese reagents towards nitrostyrenes was studied (Scheme 17). Depending on the exact reaction conditions, products of 1,4-addition **44**, reductive dimerization **45**, and/or the cross-coupling products **3** prevailed (Scheme 17). The formation of the latter was justified by a 1,2-addition-migration-elimination sequence (Scheme 1, path B). 

## 4. Nitrostyrenes in Denitrative C(sp^2^)-Heteroatom Cross-Couplings

**Scheme 18:**
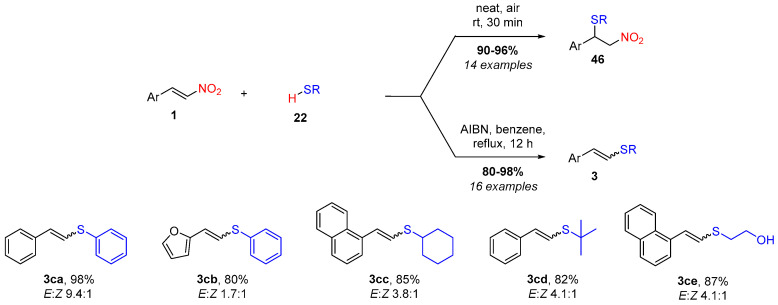
Reactivity of mercaptanes with nitrostyrene under various conditions reported by Yao [38].

Apart from the direct formation of the C-C bond, the denitrative cross-couplings offer a possibility to create sigma bonds between styrenes and various heteroatoms. The most explored is the formation of C-S and C-P bonds with the heteroatom at different oxidation states. The first example of denitrative cross-coupling leading to the formation of a new C-heteroatom bond was reported in 2009 (Scheme 18) [38]. Yao investigated a reaction of nitrostyrenes **1** with thiols **22**, under various reaction conditions. Depending on the exact reaction set-up, two different reaction products or their mixtures were formed. Under solvent-free conditions at room temperature, the conjugate addition occurred, and adducts **46** were isolated in excellent yields. However, when the reagents were combined in refluxing benzene in the presence of AIBN, a completely different reaction pathway was observed. A mixture of *E*- and *Z*-vinyl sulfide **3** was formed as a result of the radical addition-elimination process. Yao’s methodology is the first example where a heteroatom-centered radical (thiyl) participated in the cross-coupling of nitrostyrenes. 

**Scheme 19:**
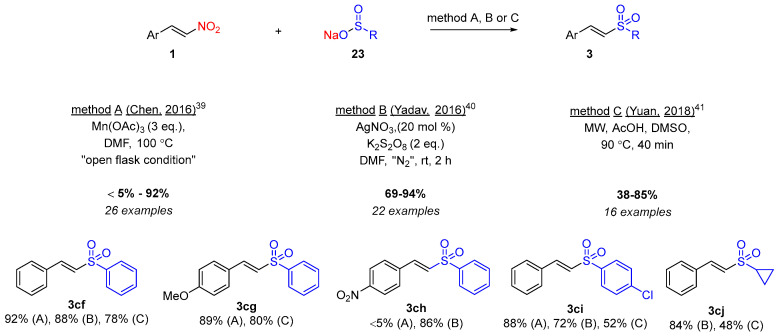
Synthesis of conjugated sulfones from nitrostyrenes and salts of arylsulfinic acids [39,40,41].

Within three years, between 2016 and 2018, several groups published closely related transformations of nitrostyrenes **1** and sodium salt of aryl sulfinic acids **23**, to form conjugated sulfones **3** (Scheme 19). In 2016, Chen reported the utilization of excess of benign manganese(III) acetate to promote the reaction in DMF at elevated temperature (Scheme 19, method A) [39]. The same year, Yadav developed a catalytic system using silver nitrate as the catalyst and potassium persulfate as co-oxidant (Scheme 19, method B) [40]. The mild conditions allowed the formation of miscellaneous conjugated sulfones **3**, including the labile styryl cyclopropyl sulfone **3cj**. Two years later, in 2018, Yuan disclosed a metal-free variant of the same transformation (Scheme 19, method C) [41]. The reaction proceeded in acetic acid at elevated temperatures under microwave irradiation. Overall, all three complementary variations of the method provide rapid access to both styryl alkyl sulfones and styryl aryl sulfones.

**Scheme 20:**
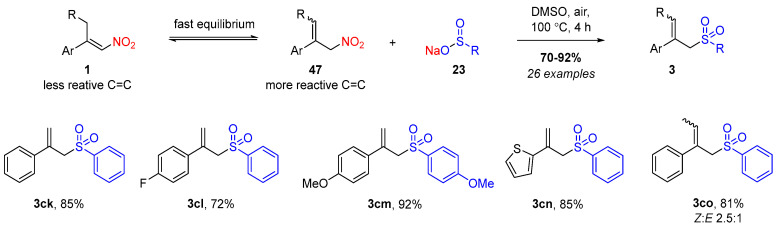
Synthesis of allylic sufones from nitrostyrenes reported by Shi and Chen [61].

In the light of these three discoveries, Shi and Chen observed a very different reaction outcome when α-substituted nitrostyrenes **1** reacted with the sodium salt of aryl sulfinic acids **23** (Scheme 20) [61]. Unlike in the previous reports, the reaction between sodium sulfinic acid salt and the nitroolefin yielded allylic sulfones instead of the conjugated sulfones. The key to the observed reactivity was the Lewis base-promoted equilibrium between nitroalkenes **1** and allylic nitro compounds **47**. The latter, according to Shi and Chen’s hypothesis, contains a C=C bond, with higher reactivity towards sulfonyl radical addition.

**Scheme 21:**
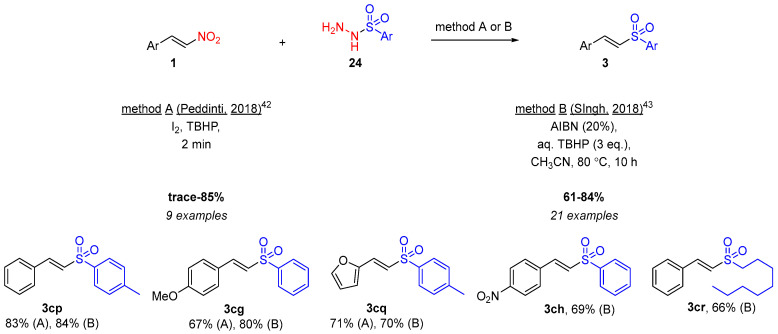
Cross-coupling of nitrostyrenes and sulfonyl hydrazides [42,43].

**Scheme 22:**
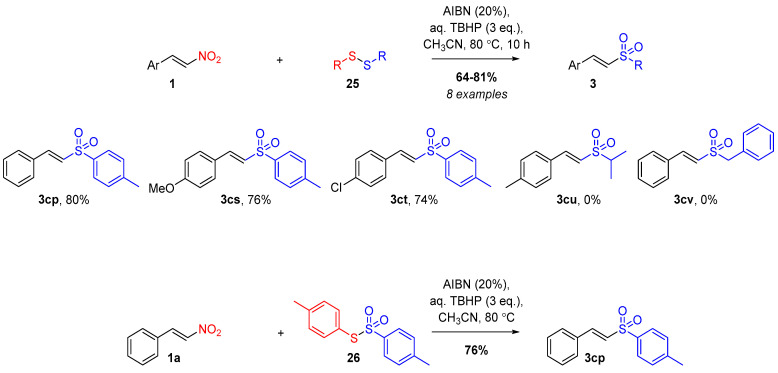
Singh’s disulfide and aryl thiosulfonate cross-couplings with nitrostyrenes [43].

A series of alternative cross-coupling partners for the preparation of conjugated sulfones from nitrostyrene was described in 2018 by Peddinti and Singh, respectively (Scheme 21 and Scheme 22) [42,43]. Firstly, Peddinti described a rapid coupling of nitrostyrenes **1** with aryl sulfonyl hydrazides **24**, promoted by iodine and TBHP (Scheme 21, method A) [42]. Based on several control experiments and literature data, Peddinti proposed that the standard radical addition-elimination reaction mechanism is triggered by the formation of sulfonyl radical from sulfonyl hydrazide. It was suggested that the established optimal conditions were more suitable for β-nitrostyrene containing electron-rich aryl moiety. The substrate scope, focusing mostly on electron-rich aryl, reflected this hypothesis. The report from Singh identified a mixture of AIBN and aqueous TBHP as the most suitable initiator of the crucial sulfonyl hydrazide decomposition to sulfonyl radical (Scheme 21, method B) [43]. A significantly broader substrate scope was reported; electron-rich and electron-poor aryl substituents in the nitrostyrene were tolerated. Aliphatic sulfonyl hydrazide and octyl sulfonyl hydrazide successfully underwent the transformation as well. 

While investigating the reactivity of sulfonyl hydrazide, Singh made another important discovery. Under the same reaction conditions, aryl disulfides **25** and aryl thiosulfonate **26** yielded identical reaction products **3** (Scheme 22) [43]. It was hypothesized that the disulfide **25** was in situ converted to thiosulfonates **26**, which then afforded sulfonyl radical and, eventually, the vinyl sulfone **3**. Feasibility of the new disulfide—nitrostyrene coupling was tested on a series of substrates, which included an aliphatic disulfide and benzyl disulfide. The latter did not form the desired products **3cu** and **3cv**.

**Scheme 23:**
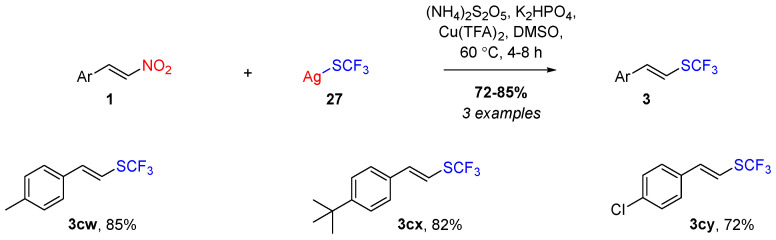
Zheng‘s synthesis of trifluoromethyl thioethers via denitrative cross-coupling [44].

A unique application of nitrostyrenes **1** in the synthesis of trifluoromethyl thioethers was reported by Zheng et al. (Scheme 23) [44]. The patented invention disclosed a method for cross-coupling of nitrostyrenes **1** with silver trifluoromethyl sulfide (**27**), under copper trifluoroacetate catalysis. Ammonium persulfate was used as an oxidant and dipotassium phosphate as an additive. The reaction occurred under the gentle reaction conditions at elevated temperature in dimethyl sulfoxide with good yields. To demonstrate the utility of the novel trifluoromethylthiolation, three examples of the substrate scope were disclosed. 

**Scheme 24:**
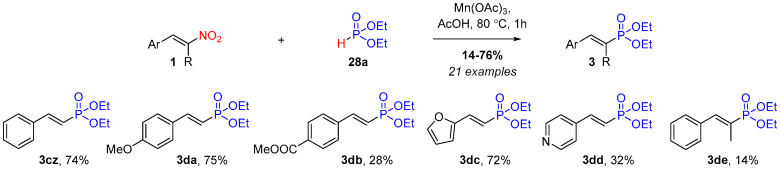
Pan’s and Zou’s denitrative cross-coupling in the synthesis of conjugated ethyl phosphonates [45].

**Scheme 25:**
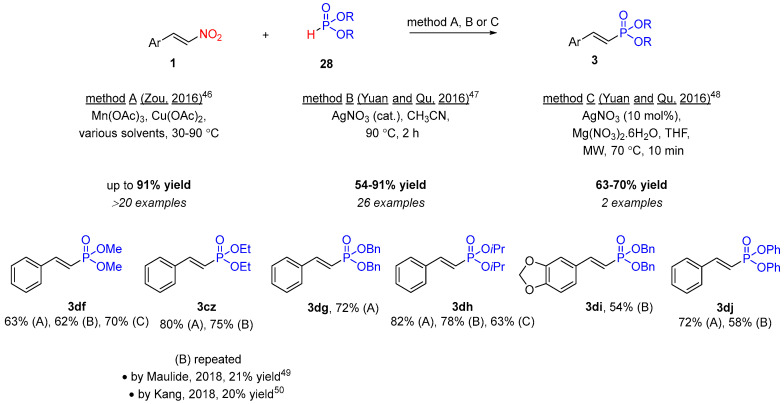
Denitrative cross coupling in the synthesis of conjugated phosphonates [46,47,48,49,50].

In parallel with the development of the cross-coupling yielding conjugated sulphones, analogous fruitful research on conjugated phosphonates was ongoing (Scheme 24 and Scheme 25). These can be traditionally accessed by various well-established methods, such as Wittig reaction or the Hirao reaction [62,63,64]. Nevertheless, the direct coupling of nitrostyrene with dialkyl phosphites certainly enhanced the existing portfolio of the method, due to its unconventional and straightforward approach. A very benign method was discovered by Pan and Zou, who used manganese(III) acetate in hot acetic acid as a gentle oxidant to promote the denitrative transformation (Scheme 24) [45]. The denitrative phosphorylation was limited only to diethyl phosphite **28a**. Pan and Zou proposed a radical addition-elimination mechanism initiated by the formation of phosphonyl radical by oxidation of the P-H bond in the diethyl phosphite (Scheme 1, path A). The breakthrough discovery inspired several similar reports which followed in the next few years.

Zou et al., in a patent from 2016, altered Pan’s and Zou’s conditions and significantly expanded the scope and utility of the transformation, with examples of various alkyl and aryl phosphites **28** as cross-coupling partners (Scheme 25, method A) [46]. Zou retained manganese acetate as the oxidant, but replaced acetic acid for various organic solvents (MeOH, *i*PrOH, CHCl_3,_ CH_3_CN, dichloroethane, AcOH, EtCOOH, toluene), and doped the system with copper acetate. His modified reaction conditions facilitated the preparation of conjugated phosphonates **3** previously inaccessible by this method. Methyl, ethyl, propyl and benzylesters and even phenyl phosphonates were readily accessed in short reaction times (compounds **3df**, **3cz**, **3dg, 3dh** and **3dj**). An alternative strategy for the generation of P-centered radicals from dialkyl phosphite was identified by Yuan and Qu (Scheme 25, method B) [47]. More expensive silver nitrate was used in a catalytic amount (15 mol %). Yuan and Qu also reported further modification of the silver nitrate-containing system, taking advantage of the microwave irradiation (AgNO_3_, Mg(NO_3_)_2_.6H_2_O, THF, 70 °C, MW, Scheme 25, method C) [48]. The benefits of the direct preparation of conjugate phosphonates from nitrostyrene we re soon after the discovery appreciated by two research groups. Maulide [49] and Kang [50] independently in 2018 repeated the reaction of nitrostyrene and diethyl phosphite and demonstrated its utility in a straightforward synthesis of conjugated phosphonate **3cz** (Scheme 25). Albeit, **3cz** was obtained in low yield, the simplicity and reliability of the novel approach prevailed over the more traditional preparations of conjugated phosphonates. 

## 5. Nitrostyrenes in Denitrative C(sp^2^)-C(sp^2^) Cross-Couplings

Traditional and the most popular transition-metal catalyzed cross-couplings, such as Suzuki, Heck, or Stille reactions, enable the formation of a sigma bond between two sp^2^-hybridised carbon atoms. First examples of analogous denitrative couplings of nitrostyrene have become available only in the last decade and have been far less frequently used. However, the increasing number of suitable coupling partners for nitro olefins and the intense research on the emerging field hold promises for future cross-coupling development. Considering the easy access to nitroolefins, their denitrative coupling has the potential to become a complementary tool to the Nobel-prize awarded reactions. 

**Scheme 26:**
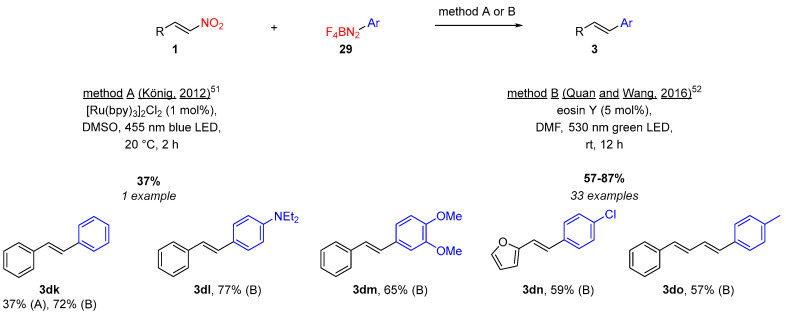
Denitrative cross-coupling of nitrostyrenes and diazonium salts in the synthesis of stilbenes [51,52]

**Scheme 27:**
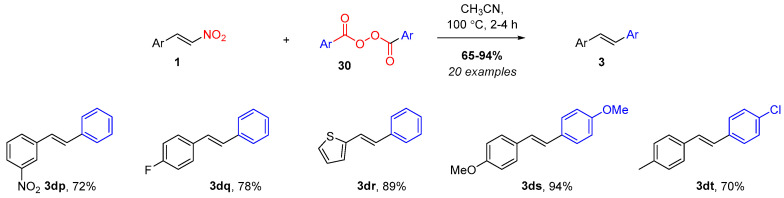
Singh’s synthesis of stilbenes via denitrative cross-coupling of nitrostyrenes and aryl peroxides [53].

**Scheme 28:**
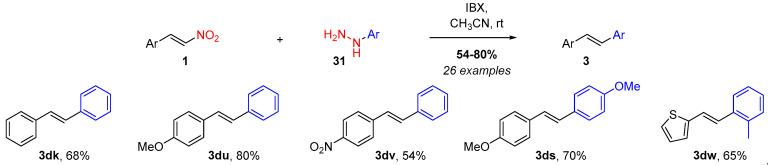
Akamanchi’s utilization of arylhydrazines in the cross-coupling with nitrostyrenes [54].

König described the very first example of denitrative cross-coupling of nitrostyrene with benzene diazonium salt **29** in 2012 (Scheme 26, method A) [51]. The work dedicated mostly to the cross-couplings of arene diazonium **29** with styrenes also provided crucial proof of principle for the coupling with nitrostyrenes **1**. The transformation was performed under very mild photochemical conditions, with only the single example of the ruthenium-catalyzed coupling being disclosed. The scope of the photochemical transformation was later significantly expanded by Quan and Wang, who demonstrated its utility in the synthesis of a broad range of *trans*-stilbenes (Scheme 26, method B) [52]. Quan’s and Wang’s protocol relied on the visible-light-induced and transition-metal free photochemical processes. According to the proposed mechanism, the aryl diazonium salts were converted to aryl radicals, which then reacted in the standard radical addition-elimination sequence (Scheme 1, path A). In the following years, *trans*-stilbenes became highly popular target compounds for cross-coupling of nitrostyrenes with various readily available organic compounds. Singh and Akamanchi developed new syntheses of stilbenes, using organic aryl peroxides and aryl hydrazines, respectively (Scheme 27 and Scheme 28) [53,54].

Singh generated the reactive phenyl radical from *tert*-butylperoxybenzoate (TBPB) or benzoyl peroxide (BPO) (Scheme 27) [53]. The in situ thermally generated reactive intermediate was used for the coupling with a range of β-nitrostyrenes. Analogously to the utilization of diazonium salts, Singh’s methodology relied on the simultaneous generation of aryl radical and low energy small molecules during the programmed decomposition of the highly reactive starting material. The method, proceeding via the radical addition-elimination pathway (Scheme 1, path A), is limited to organic aroyl peroxides. 

An alternative synthesis of stilbenes from nitrostyrene was disclosed by Akamanchi, who generated the key aryl radical from a range of aromatic hydrazines **31** and IBX (Scheme 28) [54]. The convenient procedure enabled rapid access to both symmetrical and unsymmetrical stilbenes.

**Scheme 29:**
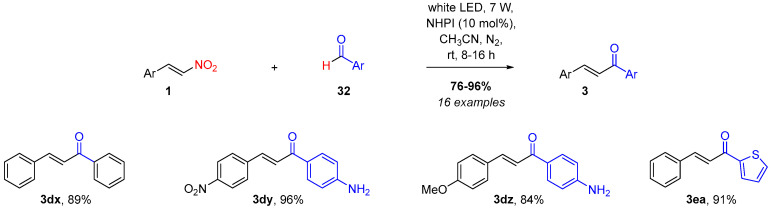
Yadav’s denitrative cross-coupling of nitrostyrenes and aromatic aldehydes in the synthesis of chalcones [55].

Chalcones, as another class of fundamental building blocks of organic chemistry, are also accessible from nitrostyrene via a cross-coupling with aryl carbaldehydes **32** (Scheme 29) [55]. Yadav’s studies in 2018 revealed that these two main building blocks of organic synthesis could be coupled efficiently under photochemical conditions. Interestingly, the radicals generated from aldehydes **32** after a photochemical cleavage of the C-H bond does not undergo decarbonylation (see Scheme 11), but rapidly reacts with the electrophilic double bond present in nitrostyrene **1** via addition-elimination mechanism, generating chalcones **3**. Broad in the range of substrates and comfortable for the actual execution, the methodology offers a new alternative to the plethora of existing chalcone syntheses. 

**Scheme 30:**
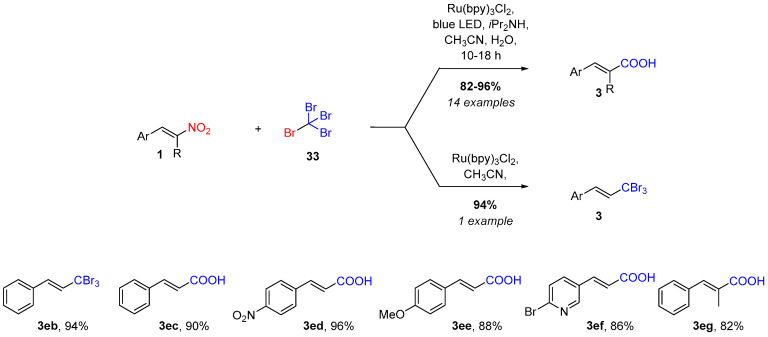
Yadav’s cross-coupling of tetrabromomethane with nitrostyrenes [56].

At the same time, Yadav discovered a rare cross-coupling of nitrostyrene with polyhalogenated alkane (Scheme 30) [56]. The approach, employing Ru-complex as a catalyst, allowed a divergent transformation of tetrabromomethane (**33**). Depending on the exact reaction conditions, either cinnamic acid or β-tribromomethyl-substituted alkene **3** was isolated from the reaction mixture. The original Ru-catalysed photochemical reaction was performed in acetonitrile, with water as a co-solvent and diisopropylamine as an additive and yielded cinnamic acids **3**. A thorough investigation revealed that the absence of water and diisopropylamine from the reaction mixture suppressed the hydrolysis and allowed the isolation of the tribromomethylated styrene **3**. A mechanistic study of both processes revealed that the initial photochemical generation of tribromomethyl radical, which underwent addition to nitro olefin, was followed by denitrative elimination. Efforts to extend the methodology to alkyl substituted nitro olefins met limited success. Altogether, Yadav’s fruitful investigation enhanced the nitrostyrene cross-couplings for three useful and unprecedented transformations. 

**Scheme 31:**
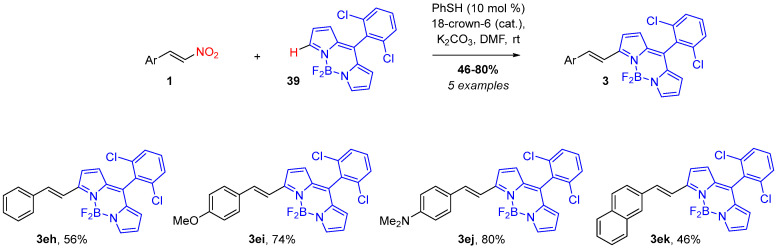
Dehaen’s vicarious nucleophilic substitution involving nitrostyrenes and BODIPY [58].

**Scheme 32:**
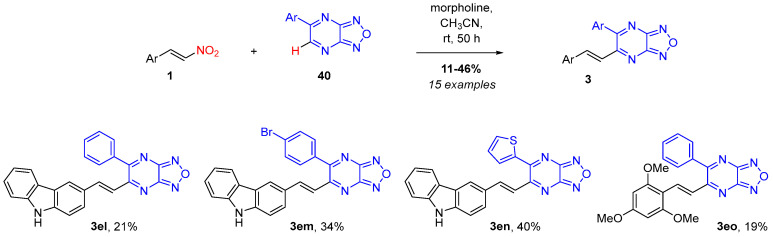
Irgashev’s vicarious nucleophilic substitution involving nitrostyrenes and pyrazine derivatives [59].

The utilization of vicarious nucleophilic substitution (VNS) in the cross-coupling of nitrostyrenes was reported by Dehaen and Irgashev (Scheme 31 and Scheme 32) [58,59]. When searching for new popular fluorescent dyes, Dehaen discovered a mechanistically intriguing transformation of boron dipyrromethene (BODIPY) **39** (Scheme 31) [58]. Thus, the treatment of BODIPY **39** with nitrostyrene **1** in the presence of a base and/or a nucleophile resulted in the formation of styryl-substituted BODIPY **3**. The overall transformation was rationalized as a result of vicarious nucleophilic transformations (Scheme 1, path C). In attempts to overcome the competing auto condensation of nitrostyrene, several other nucleophiles and bases were tested. A mixture of potassium carbonate, 18-crown-6 (catalytic amount) and thiophenol in dimethylformamide was identified as the most suitable reagent. 

Irgashev observed the same type of VNS mechanism in 2015 on pyrazine system **40** (Scheme 32) [59]. Initial attempts to utilize Dehaen’s system to promote the reaction led to the decomposition of starting materials. Consequently, Irgashev’s investigation identified morpholine in acetonitrile at an elevated temperature as the most suitable set of reaction conditions. The styrylation of several heteroarenes demonstrated the broader synthetic potential of this protocol. Variations of both aryl substituents on the nitrostyrene and the pyrazine system were tolerated, yielding conjugated polycyclic heterocyclic systems. 

## 6. Conclusions

Since Seebach’s and Russell’s groundbreaking discoveries in 1992, the reviewed stereoselective denitrative cross-couplings of nitrostyrenes have formed a new, exciting field of organic chemistry. Many easily accessible raw materials, such as various organometallics, organic iodides, alkenes, aldehydes, hydrazines, sulphinic acid derivatives and phosphites, undergo the coupling with nitrostyrenes yielding highly valuable products. The transformation offers new access to a broad range of important functionalized alkenes, including β-alkylated styrenes, chalcones, stilbenes, cinnamic acids, and conjugated sulfones and H-phosphonates. The intensified research activities in the past decade have led to the increasing number and variety of accessible products. However, the novel cross-coupling still offers only limited access to new (C)-(heteroatom) bonds, and lacks generality. Employment of state-of-the-art photochemical catalytic processes and electrochemical generation of radical species hold promises to solve this and other existing drawbacks. Without a doubt, many more efficient and unprecedented discoveries are ahead of us, as several relevant articles were published already during the preparation of this manuscript [65,66].
